# Mechanical Properties and Gamma-Ray Shielding Performance of 3D-Printed Poly-Ether-Ether-Ketone/Tungsten Composites

**DOI:** 10.3390/ma13204475

**Published:** 2020-10-09

**Authors:** Yin Wu, Yi Cao, Ying Wu, Dichen Li

**Affiliations:** 1 School of Aerospace Engineering, Xi’an Jiaotong University, Xi’an 710049, China; wyxjtu@stu.xjtu.edu.cn; 2 State Key Laboratory for Manufacturing Systems Engineering, Xi’an Jiaotong University, Xi’an 710049, China; 3 Nuclear Power Institute of China, Chengdu 610213, China

**Keywords:** shielding performance, PEEK composite materials, gamma-ray, FDM, tungsten

## Abstract

Nuclear energy provides enduring power to space vehicles, but special attention should be paid to radiation shielding during the development and use of nuclear energy systems. In this paper, novel composite materials containing poly-ether-ether-ketone (PEEK) as a substrate and different tungsten contents as a reinforcing agent were developed and tested as shielding for gamma-ray radiation. Shielding test bodies were quickly processed by fused deposition modeling (FDM) 3D printing, and their mechanical, shielding properties of composite materials were evaluated. The results revealed shielding materials with excellent mechanical properties which can further be improved by heat treatment. Under 0.45 MPa load, the heat deflection temperature of PEEK/tungsten (metal) composites was significantly lower than that of PEEK/boron carbide (non-metal) composites. The new shielding materials also demonstrated better shielding of low-energy ^137^Cs than high-energy ^60^Co. The gamma-ray shielding rates of test pieces shielding materials made of the same thickness changed exponentially with the tungsten content present in the composite materials. More tungsten led to a better shielding effect. At the same tungsten content, the gamma-ray shielding effect showed a proportional relationship with the thickness of the shielding test bodies, in which thicker test bodies induced better shielding effects. In sum, the integration of 3D printing in the mechanical design and manufacturing of shielding bodies is an effective and promising way for quick processing when considering diverse rays and complex environments. Lighter shielding bodies, at lower costs, can be achieved by structural design in limited space to maximize the material utilization rate and reduce waste.

## 1. Introduction

Space nuclear power systems offer lasting power sources for space exploration but increase the possibility of exposure to nuclear radiation for both the astronauts and electronic equipment [1,2,3]. In the 20th century, accidents caused by space nuclear power sources for both the United States and Russia have engendered irreversible damage to humans, space equipment, and the environment on earth. The radiation responsible for such damages has been gradually identified [4,5], in which gamma-rays and neutron radiation possess strong penetrating abilities, thereby deserve special attention when dealing with shielding problems. Gamma-rays generally accompany the neutron radiation produced by isotope sources or nuclear facilities, where thousands of discovered radionuclides could generate gamma-rays [6]. The exposure of the human body to gamma-ray radiation would likely result in damage at the cellar level, altering the whole body mechanism. Therefore, the development of new radiation shielding materials along with corresponding processing technologies is mandatory for the protection of astronauts from nuclear radiation.

Gamma-rays possess strong penetrating ability since they possess short wavelengths, and therefore high energy [7]. Therefore, protective materials made from high-atomic number heavy metal materials, construction materials, are usually used as effective shields against gamma-rays [8,9,10,11]. These include iron, tungsten, lead, concrete, and silicate like cement and rock with excellent shielding performances [12,13]. The most used shields are lead-based materials [14,15], where certain amounts of lead could shield initial or secondary gamma-ray radiation [16]. However, lead cannot resist high temperatures despite its elevated corrosion resistance to air oxidation and acid corrosion [17]. Creep in lead begins forming above 260 °C, validating its poor hardness and low mechanical strength. Furthermore, lead is toxic as a heavy metal pollutant [18,19,20]. After entering the human blood, lead combines with red blood cells and becomes difficult to eliminate from the body, causing irreversible damage. Therefore, the use of lead-based materials currently is restricted [21,22].

As alternatives, tungsten-nickel alloys with the high corrosion resistance to acid, alkali, aqua regia and air at normal temperatures, are excellent shielding materials against gamma-rays. Tungsten alloys are also effective materials for gamma-ray shielding due to their high hardness and one third reduced thickness compared to lead [23,24,25]. Tungsten is characterized by an elevated density, high hardness and superior melting point, making its deformation at high temperatures more difficult. Compared to lead, tungsten does not react with particles and is not toxic, thereby is better at tackling secondary toughness radiation caused by lead composite materials [26]. This also would eliminate the danger of lead-related toxicity and reduce the cost of waste disposal. Despite its high cost (13-fold more expensive than lead [27,28]), tungsten and tungsten compounds are still regarded as an ideal dielectric shielding material with excellent gamma-ray shielding ability [29,30,31]. Therefore, finding suitable processing technologies for the proper, economical, and efficient use of tungsten materials is highly desirable.

Polymer materials with high strength, superior toughness, and elevated temperature resistance are widely used in electronics, machinery, aerospace, and other industrial fields [32,33,34,35]. Thermoplastic/tungsten composite materials, such as nylon and polyamine, have close densities to that of lead but much stronger in terms of yield strength. Besides, these materials have no toxicity, and are usually produced by molding and injection molding methods [36,37]. However, errors always occur during the design processing since die-casting molds have complex shapes, curved surfaces, and narrow grooves [38,39]. The attenuation of low-energy gamma-rays by epoxy resin is mainly achieved by a photoelectric absorption effect but the irradiation accelerates the aging of epoxy resin. To limit this effect, poly-ether-ether-ketone (PEEK) as a semi-crystalline thermoplastic high-temperature polymer with an elevated melting point is widely adopted thanks to its excellent features in terms of mechanical properties and superior resistance against heat, good corrosion resistance, and relevant fatigue and wear resistance [40,41]. The PEEK irradiation aging also ensures excellent radiation resistance. However, the high melting point of PEEK may lead to elevated viscosity of viscoelastic fluid passing through the head during processing, thereby generating excess flow resistance. Nevertheless, elastic turbulence occurs at a certain extrusion speed limit, forming defects and generating distortion on the surface of the molten mass. Additionally, PEEK is largely constrained by high cost and processing difficulties [42].

With the rapid development of 3D printing of PEEK and PEEK composite materials, PEEK has widely been adopted for applications in biology, medicine, aerospace, and other fields [43,44,45,46,47]. However, only a handful of studies dealing with the nuclear radiation shielding performances of 3D-printed PEEK and PEEK composite materials have so far been reported. For instance, 3D printing technology could form materials layer by layer based on computer aided design (CAD) model, thereby allowing better design and processing according to “creative-design-3D-printing-product-application” [48,49,50]. The 3D printing technical committee of the American Society for Testing and Materials (ASTM) divides 3D printing technology into seven categories [51,52], among which fused deposition manufacturing (FDM) is favored due to its simple operation and fast molding speed.

Under extreme space conditions, lightweight structures should have ideal mechanical and shielding performances, which cannot be achieved by traditional machining. In this paper, new gamma-ray shielding materials were prepared by FDM 3D printing process using PEEK as substrate and tungsten powders as a reinforcing agent. The mechanical and shielding properties of the obtained materials were examined. The material composition, multi-layer structure, and hetero-structure shielding body were all optimized. The results suggest that FDM 3D printed shielding bodies have excellent mechanical properties and shielding characteristics against various radiations. More importantly, the use of materials was maximized combining both lightweight and cost-saving mediums.

## 2. Experimental

### 2.1. Materials

**Figure 1:**
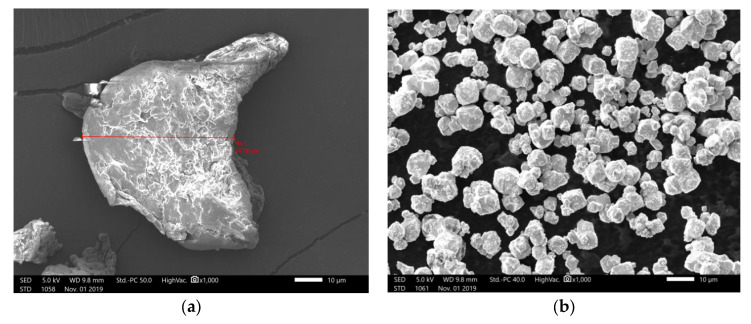
Microstructures of poly-ether-ether-ketone (PEEK) under electron microscopy: (**a**) PEEK and (**b**) tungsten.

Tungsten powders of different weights (G50 = 1 μm, density 19.35 g/cm^3^, Qinghe County Xindun Metal Material Co., Ltd., Hebei, China) and PEEK (G50 = 50 μm, density 1.30 g/cm^3^, VICTREX^®^, 450PF, Lancashire, UK) were used for the preparation of PEEK/tungsten composite materials, in which PEEK was employed as substrate and tungsten as a reinforcing agent. Composite materials with different tungsten contents (50 wt. %, 60 wt. %, and 70 wt. %) were obtained after vacuum drying, materials mixing, air flow dispersion, screw granulation, and wire extrusion processes. The respective electron microstructures of PEEK and tungsten are shown in Figure 1a,b. A certain agglomeration was noticed in tungsten.

### 2.2. Preparation of FDM 3D Printed PEEK/Tungsten Composite Materials

**Figure 2:**
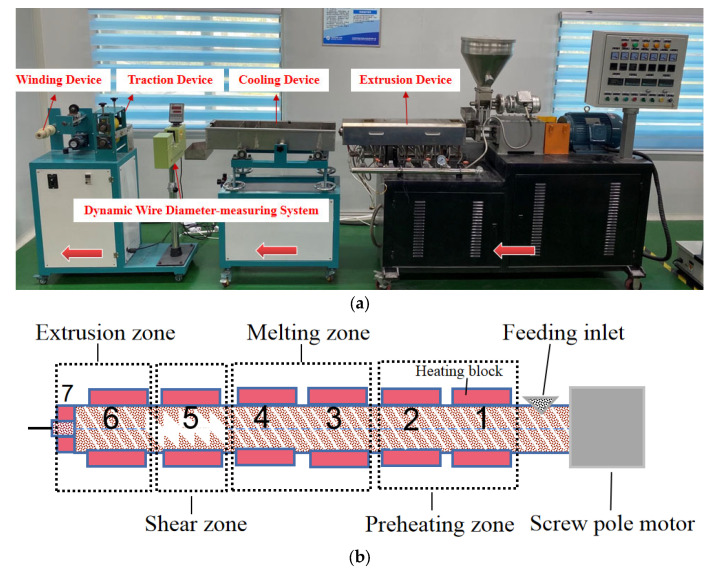
Preparation process of PEEK and PEEK composite fused deposition modeling (FDM) 3D printing shielding wire: (**a**) a picture of the screw extruder equipment, (**b**) the heating zones of the extrusion device.

PEEK/tungsten composites powders were prepared by a screw extruder according to the requirement of fused deposition modeling (FDM) 3D printing (Figure 2). A picture of the screw extruder equipment (YTG-20 Twin Screw Extruder, Guangzhou Yongtuo Plastic Machinery Co., Ltd., Guangzhou, China) was provided in Figure 2a. The dark red arrows referred to the operation direction from right to left. The four light red arrows corresponded to the extrusion device, traction device, cooling device, and winding device. A total of seven heating zones existed in the extrusion device. The temperature from right to left was set at 310 °C, 325 °C, 340 °C, 345 °C, 345 °C, 330 °C, 320 °C. The rotation speed of the screw and traction speed were set according to the wire diameter requirement of FDM 3D printing. During experiments, the rotation speed of the screw was set to 32 r/min and the traction speed was 8 m/min. Figure 2b demonstrated the exact places where the different heating zones were located.

As can be seen from Figure 2b, in the extrusion device, there were four zones with seven heating blocks controlling the temperature of each heating block through thermocouples. These four zones were preheating zone, melting zone, shear zone and extrusion zone. The corresponding temperature from 1 to 7 is set as 310 °C, 325 °C, 340 °C, 345 °C, 345 °C, 330 °C, and 320 °C. The heating block 1 and 2 constituted the preheating zone where PEEK and PEEK composite materials were transported for preheating. During the preheating process, the PEEK composite material underwent a transition from a glass state to a high elastic state, then to a viscous fluid state. Afterwards, the PEEK and PEEK composite materials were pushed into the melting zone composed of heating block 3 and 4 for completely melting. The completely melted PEEK and PEEK composite materials were sheared and extruded through heating block 5 in the shear zone, so that the air generated in the barrel could be removed. The sheared and stable PEEK and PEEK composite materials finally flowed out through the extrusion zone consisting of heating block 6 and 7.

The material ratios of the composite materials were provided in Table 1. PEEK/tungsten composite materials with respective tungsten mass ratios of 50 wt.%, 60 wt.%, and 70 wt.% were obtained after full dispersion. The corresponding tungsten volume ratios were estimated as, respectively, 6.30 vol%., 9.15 vol%., and 13.5 vol%. The samples #0, #1, #2 and #3 referred to pure PEEK, 50 wt.%, 60 wt.% and 70 wt.% PEEK/tungsten composite materials, respectively.

**Figure 3:**
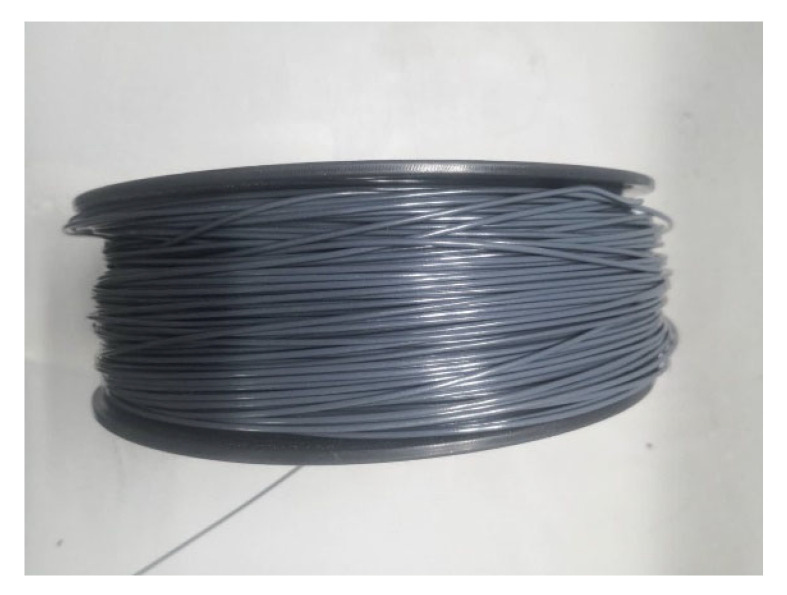
FDM 3D printed special PEEK/tungsten shielding materials.

Novel composite materials suitable for FDM printers were obtained after composite material dispersion, twin-screw granulation, and single-screw extrusion processes. The diameter of the composite material wire was estimated to 1.75 mm (±0.05), thereby meeting the FDM processing requirement (Figure 3).

The compactness values of PEEK and PEEK composites measured by the drainage method are listed in Table 2. Both PEEK and PEEK composite materials displayed good compactness. The sample #0 represented pure PEEK, and samples #1, #2 and #3 referred to 50 wt.%, 60 wt.% and 70 wt.% PEEK/tungsten composite materials, respectively.

According to Table 2, the wire compactness of sample #0 was 100%. The wire also looked to be free of bubbles and defects. The addition of tungsten led to a decline in compactness (#0 > #1 > #2 > #3), as well as a serious agglomeration in tungsten powders (G50 = 1 μm). In turn, material agglomeration should highly affect the compactness of FDM wire.

### 2.3. Dispersion of New Shielding Materials

**Figure 4:**
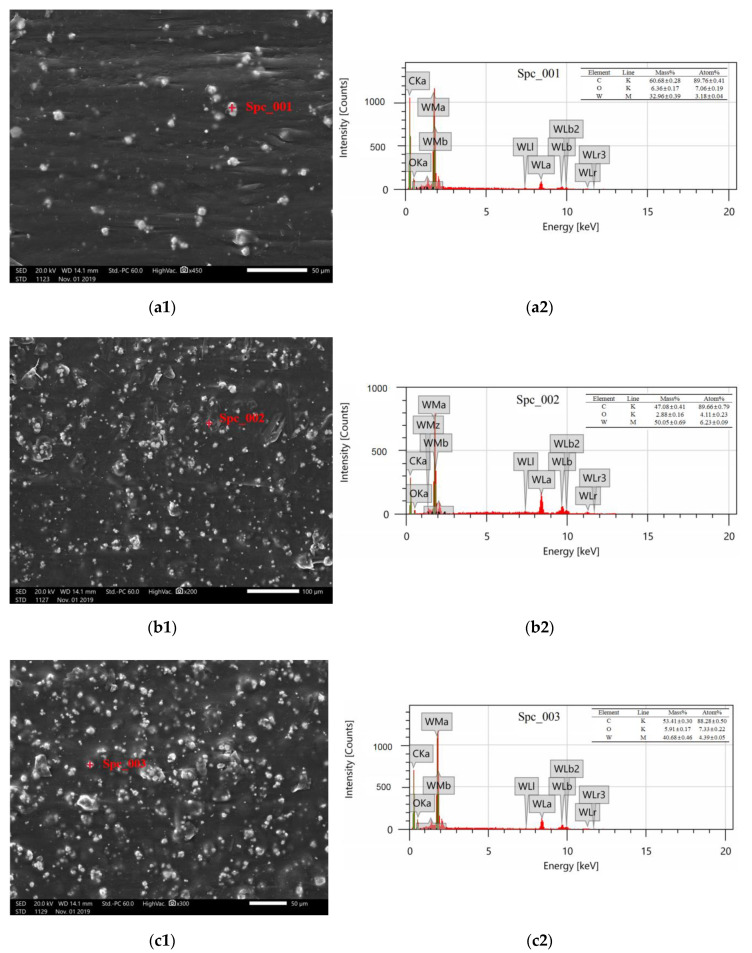
Microscope electron micrographs and EDS energy spectra of PEEK/tungsten composite materials: (**a1**) electron micrograph of #1, (**a2**) EDS energy spectrum of #1, (**b1**) electron micrograph of #2, (**b2**) EDS energy spectrum of #2, (**c1**) electron micrograph of #3, and (**c2**) EDS energy spectrum of #3.

To evaluate the dispersion of tungsten in the new shielding materials, energy dispersion spectrum (EDS) analyses were performed on the wire microstructure of FDM PEEK/tungsten composite materials by an analytical scanning electron microscope (JSM-IT500LA, JEOL Ltd., Tokyo, Japan). The characteristic X-rays emitted by different elements have different probabilities, namely different energies. EDS could quickly qualitatively and quantitatively analyze the elements in the test samples by checking the energy of photons. The microstructures of PEEK composites containing different tungsten contents were illustrated in Figure 4. Note that EDS analyses were conducted at the probe (red dots) shown in Figure 4a1,b1,c1. The probe (red dot) was used to qualitatively or quantitatively analyze the specific parts of the sample. The red dots in 4a1, b1, c1 was the position of the probe in the designated point in the sample while 4a2, b2, c2 were the qualitative and quantitative element analysis performed by the probe at the specified point in the sample.

Figure 4a2,b2,c2 clearly presented the element composition, line system, element mass percentage, and atomic percentage at the red dotted point position of the sample in Figure 4a1,b1,c1. For example, in Figure 4a2, the semi-quantitative analysis of the K-line system manifests that the sample contained C and O elements, and W element was also discovered from the semi-quantitative analysis of the M-line system. The mass content of C was 60.68%, that of O was 6.36%, and that of W was 32.96%, indicating that the white part of the sample at the 4a1 point was tungsten embedded in PEEK. The distance between tungsten and probe can be approximated by the value of tungsten content. It could also be judged the white parts in Figure 4b1,c1 represented tungsten. The dark gray parts referred to molten and solidified PEEK. The tungsten particles were evenly embedded within the PEEK substrate, and agglomeration worsened as tungsten content increased. As a result, composite materials with reduced compactness were obtained.

### 2.4. High-Temperature FDM 3D Printer

**Figure 5:**
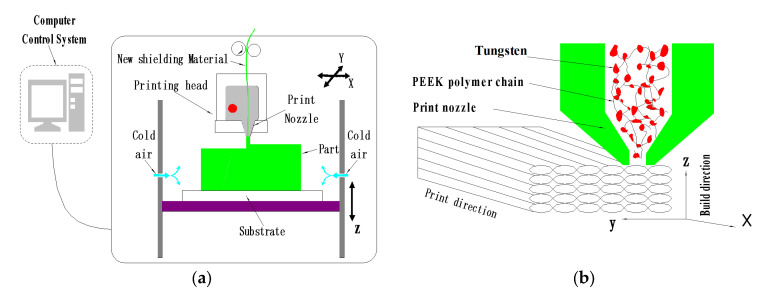
The mechanism framework and working principles of FDM: (**a**) FDM structure framework and (**b**) FDM printing and processing.

The thermogravimetry analyses determined the heat decomposition temperature of PEEK at 550 °C. The long-time high temperature significantly decreased the accuracy of the electrical and structural determination systems, thereby restricting the applications of FDM 3D printers. A picture representing the high-temperature FDM 3D printer independently developed by Xi’an Jiaotong University was displayed in Figure 5a. Note that the design adopted a series of self-developed technologies, including multi-level heating, cold water circulation, aerospace-grade heat insulation, high-temperature nozzle, and continuous printing after power-off. These features helped the realization of long-time continuous and stable processing of PEEK and PEEK composite materials. The FDM printer mainly consisted of three systems: a computer control system (data processing system), an air circulation system (cooling system), and a micro-environment control system (printing nozzle). The processing principles of FDM 3D printed PEEK composite materials were provided in Figure 5b. In this process, PEEK/tungsten composite materials will first be melted by the heating system in the nozzle. The print head will then move along the cross-sectional contour and filling trajectory. Meanwhile, the molten materials will be extruded and quickly solidifying through the cooling system to bond with the surrounding materials. During the whole process of FDM 3D printing, the microenvironment system of the molded parts would play a key role in the quality of the formation process. The printing nozzle as a heating terminal would directly be exposed to the molding materials. Additionally, the temperature of the printing nozzle will directly affect the initial temperature and cooling crystallization of the molten wire.

### 2.5. Mechanical and Shielding Properties

#### 2.5.1. Tensile and Flexural Properties Test Method

**Figure 6:**
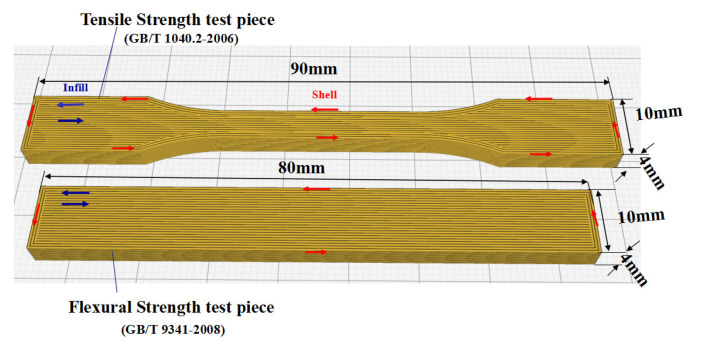
Dimensional requirements for mechanical properties of test pieces.

Test pieces were made by FDM 3D printer according to GB/T 1040.2-2006 and GB/T 9341-2008 standards [53,54]. Note that GB/T 1040.2-2006 is one of the national standards of the People’s Republic of China equivalent to ISO527-2:1993, specifying the test methods of tensile properties. According to this standard, all samples surfaces should be free of visible cracks, scratches, and other defects. Besides, burrs should be removed by sanding. Similarly, GB/T 9341-2008 was the national standard of the People’s Republic of China and equivalent to the ISO 178:1993 standard, specifying the test methods for determining the flexural properties of plastics under specified conditions. Here, the tests were conducted on two freely supported ends and in the form of central loading (three-point loading testing). The specific dimensions of test pieces measured by FDM 3D printing to determine the tensile and flexural strengths are shown in Figure 6. Five specimens were used in the property test of PEEK and PEEK composite materials, and the average value was obtained after the experiment.

The test pieces of PEEK composite materials containing different tungsten contents were processed by FDM printer and detailed dimensions were gathered in Figure 6. The test pieces were prepared by first producing the outer layer contour, in which the red arrow referred to the direction of the outer contour nozzle movement and blue arrow depicted the nozzle solid line filling direction. Furthermore, 100% solid filling was conducted during the filling process with the specific processing parameters listed in Table 3.

**Figure 7:**
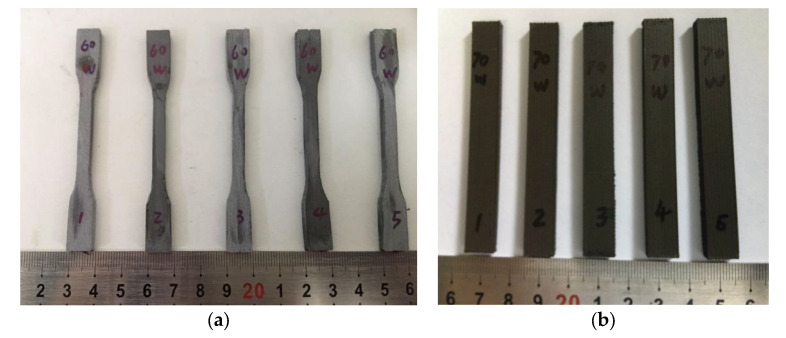
FDM 3D printed test pieces used for mechanical properties testing: (**a**) tensile strength and (**b**) test pieces for flexural strength.

The thermoplastic characteristics of PEEK induced by its high melting point (343 °C) led to larger melt viscosities after melting. However, PEEK displayed enhanced shrinkage rate and semi-crystallization after cooling. Note that tungsten had a melting point of 3410 °C combined with a small expansion coefficient and elevated thermal conductivity above 100 °C. PEEK/tungsten composite materials prepared by traditional injection molding and molding methods were often characterized by low material utilization rate, high cost, many processing defects, and difficult processing. On the other hand, the FDM 3D printing temperature control system would play an important role in successful processing. During the printing process, the selection of appropriate printing speed (nozzle movement speed), nozzle temperature, and cooling efficiency at the nozzle would largely affect the molding quality. Thus, optimal combination was utlized to complete the processing, and the as-obtained FDM test pieces employed for mechanical properties testing are provided in Figure 7.

The FDM 3D printed test pieces showed no distortion, with surfaces perpendicular or horizontal to each other. Additionally, no scratches or defects were observed on the surface, thereby fully meeting the testing requirement.

#### 2.5.2. Heat Deflection Temperature Test Method

**Figure 8:**
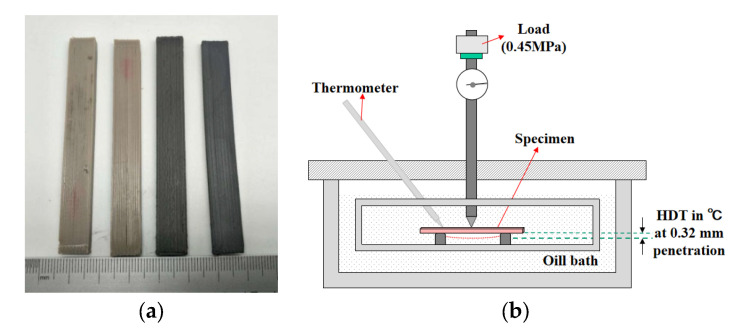
Heat deflection temperature (HDT) by Vicat: (**a**) Test pieces for HDT and (**b**) deformation and vicat softening temperature tester.

Plastics, hard rubber, and long fiber reinforced composites were used to conduct experiments according to “The Plastics-Determination of Temperature of Deflection under Load-Part 2: Plastics, Ebonite and Long-reinforced Composites” of national standard GB/T1634.2-2004 of the People’s Republic of China [55]. Equivalent to ISO 75-2:2003 (IDT), this standard specifies the detailed requirements for meeting for experiments, in which 0.45 MPa flexural stress method along with laid flat samples (size 80 mm × 10 mm × 4 mm, equivalent to flexural strength sample) were adopted for experimentation (Figure 8a). Qualified FDM 3D printed and processed test pieces were made according to GB/T 1634.2-2004 standard and tested by The Deformation and Vicat Softening Temperature Tester (Vicat/HDT-TESTER, COESFELD, Dortmund, Germany). Under 0.45 MPa load, the temperature rised from room temperature (23 °C) at the rate of 2 °C/min. When the deformation reached 0.32 mm, the corresponding oil bath temperature was the heat deflection temperature to be measured (Figure 8b).

#### 2.5.3. Gamma-Ray Shielding Test Method

**Figure 9:**
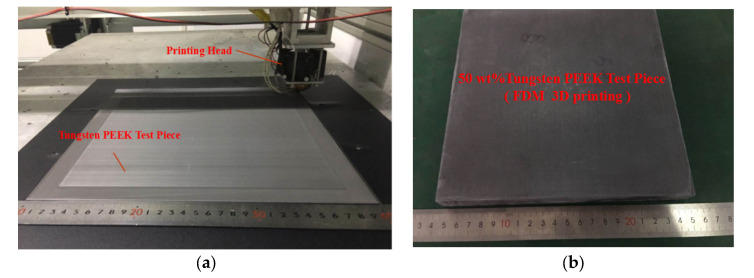
Processing of an FDM 3D printed shielding test piece: (**a**) during production and (**b**) after production.

The FDM 3D printed shielding bodies were made of solid filled test pieces (Figure 9). To evaluate the shielding effects of test bodies against gamma-rays, parameters like linear attenuation coefficient, half absorption thickness, and shielding rate of PEEK/tungsten composite materials were all measured.

**Figure 10:**
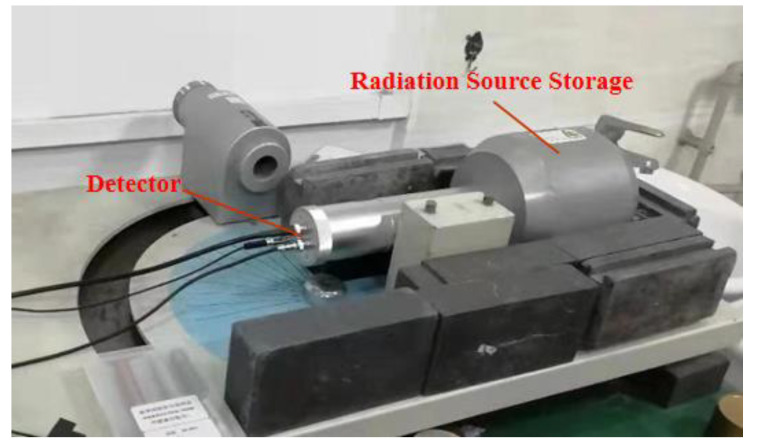
A picture showing the gamma-ray absorption experiment platform.

Two types of gamma-ray radiation sources: ^60^Co (energy 1.17 MeV with emission probability of 99.85%, 1.33 MeV with emission probability of 99.98%) and ^137^Cs (energy 0.662 MeV with emission probability of 85.10%) were employed in the experiments [56]. Since ^60^Co possessed two energy levels of 1.17 MeV and 1.33 MeV, its average energy was estimated to be 1.25 MeV. Shielding test bodies with different tungsten contents and thicknesses were irradiated by both energy sources. Figure 10 displays the experimental platform used for gamma-ray absorption, where data were collected through an NaI (Tl) detector and an integrated energy spectrometer.

**Figure 11:**
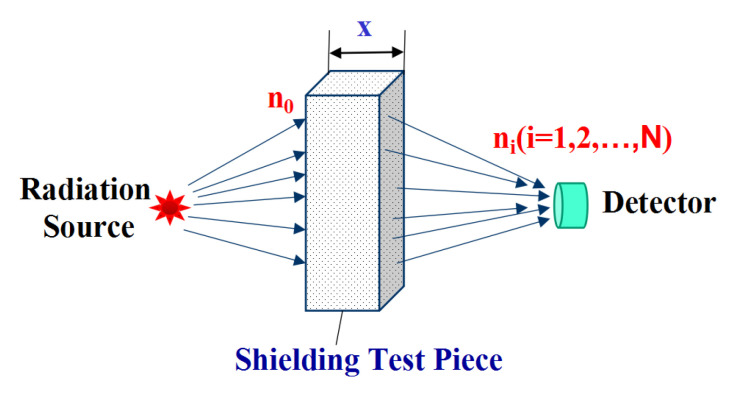
Schematic diagram of the experimental model used for gamma-ray attenuation.

The exponential decay law of gamma-rays was discovered in 1909 by Soddy and Russell [57]. A schematic diagram of the experimental model used for gamma-ray attenuation was presented in Figure 11. After the passage of gamma-rays with n_0_ radiation through the shielding test body with thickness x, the attenuated radiation amount n_i_ (i = 1, 2, …, N) could directly be described by Equation (1).

The natural logarithm on both sides of Equation (1) would yield Equation (2), reflecting the linear relationship between lnn_i_ and lnn_0_. Note that μ is called the linear absorption coefficient.

n_i_ = n_0_ × e^−μx^    (i = 1, 2, …, N) (1)

ln (n_i_) = ln (n_0_) − μx (i = 1, 2, …, N) (2)

where n_0_ is the ray intensity without shielding, n_i_ represents the ray intensity after the i-th penetrating the shielding test body, x is the thickness of shielding test body, and μ denotes the linear absorption coefficient reflecting the gamma-ray radiation energy absorbed by the shielding body.

Since the concept of range in matter cannot be applied to gamma-rays, when the gamma-ray intensity was reduced by half, the corresponding thickness of the absorbing substance was called the half-weakening layer thickness of the substance. The absorption coefficient can be expressed by the half-absorbing thickness d_½_ (Equation (3)).

(3) d12=ln2μ= 0.693μ   

The shielding effect can be expressed more vividly through the shielding efficiency (E%) presented in Equation (4).

(4) $$E\left(\%\right)=\frac{n_{0}-n_{i}}{n_{0}}\times 100\%\;\;\;\;\left(i=1,\;2,\;\cdots ,N\right)$$

where n_0_ is the ray intensity without shielding and n_i_ represents the ray intensity after the i-th penetrating the shielding test body.

## 3. Results and Discussion

### 3.1. Mechanical Properties

#### 3.1.1. Tensile and Flexural Properties

**Figure 12:**
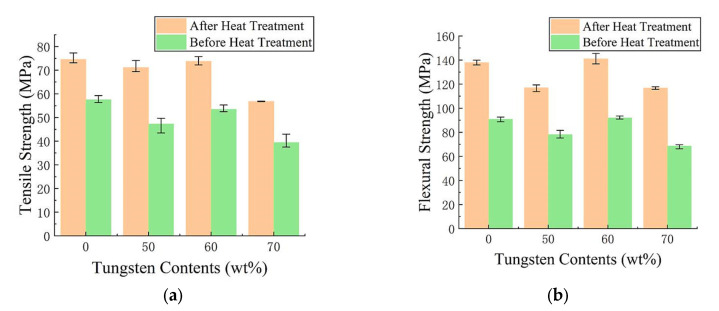
Test results of tensile and flexural strengths of PEEK and PEEK/tungsten composites: (**a**) tensile strength and (**b**) flexural strength.

Qualified FDM 3D printed and processed test pieces were made according to GB/T 1040.2-2006 and GB/T 9341-2008 standards, then tested by a computer-controlled electronic universal testing machine (GMT4304, MTS^®^, Shang Hai, China). The test results of the mechanical properties are shown by the bar graphs in Figure 12.

The tensile strength values of PEEK and PEEK composites containing different tungsten contents are provided in Figure 12a, where the green bar graphs presented the tensile strength before heat treatment used for analysis. The test speed was set at 1 mm/min. Using tungsten contents from 50 wt.% to 60 wt.%, the tensile strengths showed a slight increase. After the complete melting of PEEK to form colloids, small amounts of tungsten aggregated in the colloid with supporting roles. Under the action of wire thrust, the melted PEEK colloids became wrapped on the surface of tungsten, irregularly filling the shaped voids. After cooling and hardening of PEEK, tungsten became completely embedded and combined with PEEK to form a novel composite. At tungsten contents from 50 wt.% to 60 wt.%, the tensile strength increased by 11.59%. On the other hand, the agglomeration of tungsten became increasingly serious as tungsten powder content rose. The rapid cooling and molding of melted PEEK led to difficult infiltration of the molecular chains into the tungsten agglomerates within short periods. Therefore, the agglomerates maintained their original random accumulated gap states, and micro-pores easily formed in the cooled and molded composite materials. Tungsten contents in PEEK above 60 wt.% led to declining trends. For instance, the tensile strength of 70 wt.% PEEK/tungsten composite was 26.30% lower than that of 60 wt.% PEEK/tungsten. Additionally, the agglomeration of tungsten powders formed numerous micro-pores, highly affecting the tensile strength of the test pieces.

The flexural strength values of PEEK composites with different tungsten contents are illustrated in Figure 12b, where the green bar graphs presented the flexural strength before heat treatment used for analysis. During testing, the two ends were freely supported and the center was loaded with the test at the speed of 1 mm/min. The PEEK/tungsten composite test pieces looked bent but did not break, confirming good toughness. The addition of tungsten at contents between 50 wt.% and 60 wt.% led to a slight increase. However, the curve decreased for tungsten contents above 60 wt.%. Compared to the 50 wt.% PEEK/tungsten composite, the flexural strength of the 60 wt.% PEEK/tungsten composite material incremented by 15.10%. However, the flexural strength of the 70 wt.% PEEK/tungsten composite reduced by 25.44% when compared to the 60% PEEK/tungsten composite material. Similar to tensile strengths, the flexural strengths of the test pieces were influenced by the micro-pores caused by the agglomeration of tungsten powders. To improve the mechanical properties of the composite materials, the test pieces were thus heat-treated.

**Figure 13:**
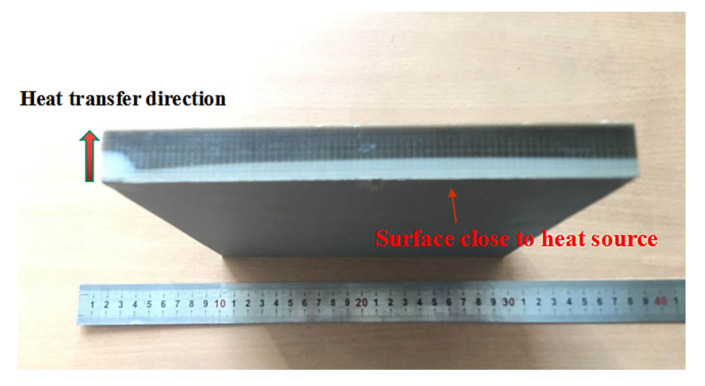
Gradient delamination of the base material PEEK FDM test piece during the heat transfer process.

The pink bar graphs in Figure 12 refer to the tensile and flexural strengths after heat-treatment, which looked to be greatly improved. Note that heat-treatment was used to fix the FDM 3D printed test pieces to fit with the standards GB/T 1040.2-2006 and GB/T 9341-2008 through fixtures in two aluminum flat plates with smooth surfaces. To fully dry the PEEK and PEEK/tungsten composite materials, the test pieces and fixtures were subjected to 80 °C heat for 5 h and then quickly placed in a 300 °C thermostatic temperature control box for 2 h. Afterwards, they were taken out and the temperature was rapidly reduced to below 140 °C, representing the crystallization temperature of PEEK. The specimens were finally cooled down for 1 to 3 days at room temperature. According to the results, the tensile strengths of pure peek, 50 wt.%, 60 wt.% and 70 wt.% PEEK composites rose by 22.88%, 33.51%, 27.52% and 30.60%, respectively. Additionally, the flexural strengths enhanced by 34.15%, 33.28%, 34.81% and 41.29%, respectively. On the other hand, PEEK as a semi-crystalline high-performance special engineering plastic largely relies on its crystallinity [58,59]. Hence, the external PEEK of the test body recrystallized when the temperature rose to 300 °C due to temperature changes. Furthermore, the unit cell preferentially grew into a crystal plane, resulting in rearranged molecular (or atoms) species of the randomly arranged molecular segments in the outer layer of the crystal cell. This turned disordered areas into regular crystalline areas. A high-crystallinity region was formed through heat transfer in the outer layers of the test pieces along with a gradient function sandwich structure in the low-crystallographic region of the inner layer. A picture of the gradient layered structure of FDM PEEK test piece (200 mm × 200 mm × 20 mm) formed by heat transfer is displayed in Figure 13. The heat transferred from one side to the other side close to the 300 °C heat source, demonstrating obvious discoloration after 0.5 h and showing the formation of two distinct areas.

**Figure 14:**
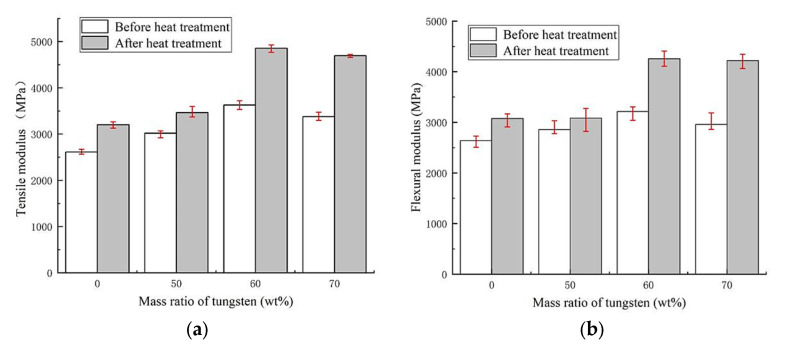
Tensile modulus and Flexural modulus of PEEK and PEEK/tungsten composites: (**a**) tensile modulus and (**b**) flexural flexural modulus.

The tensile modulus and flexural modulus of PEEK and PEEK/tungsten composites are summarized in Figure 14. Before heat treatment, the tensile modulus of PEEK composites increased with tungsten content before reaching 60%. After that, the tensile modulus started to decrease, similar to the variation trend of the flexural modulus. After heat-treatment, both the tensile modulus and flexural modulus of PEEK and PEEK/tungsten composites greatly improved. The tensile moduli of PEEK and 50 wt.%, 60 wt.% and 70 wt.% PEEK/tungsten composites all rose by 22.58%, 14.76%, 33.82%, 38.95%, respectively. The flexural moduli also enhanced by 16.54%, 7.99%, 32.41% and 42.51%, respectively.

It can be seen in Figure 12a,b and Figure 14a,b that compared to that of PEEK, the properties of 50 wt.% PEEK/tungsten composites have been slightly changed. Both the tensile strength and flexural strength of 50 wt.% PEEK/tungsten composites were slightly decreased (Figure 12a,b). As for the tensile modulus and flexural modulus, those of 50 wt.% PEEK/tungsten composite materials increased slightly (Figure 14a,b).

**Figure 15:**
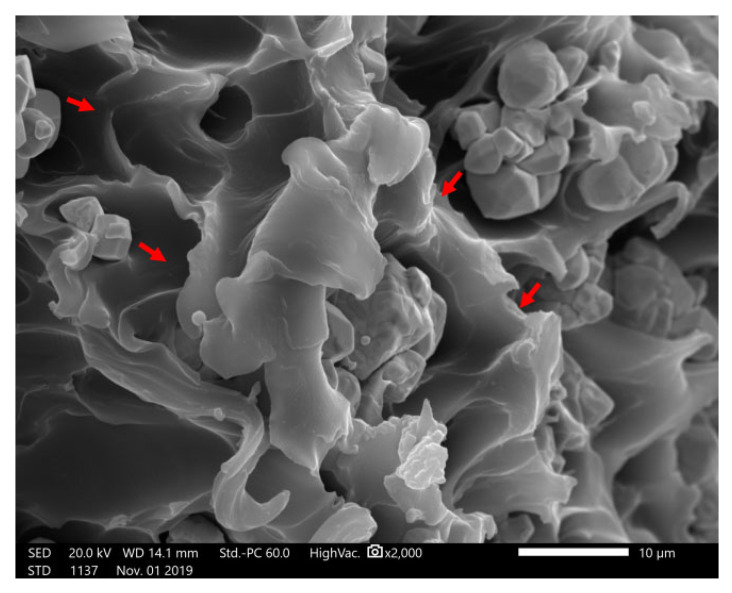
Electron micrograph of the fracture surface of 70% PEEK/tungsten composite (×2000).

The broken section of the tensile test piece made of 70 wt.% PEEK/tungsten composite was observed by an analytical scanning electron microscope (JSM-IT500LA, JEOL Ltd., Japan). As shown in Figure 15, the tungsten was nicely embedded in PEEK. Additionally, tungsten agglomerated with obvious voids (red arrow) noticeable at the fracture caused by the decrease in tensile and flexural modulus (Figure 15).

#### 3.1.2. Heat Deflection Temperature (HDT)

In areas where people are likely to be exposed to nuclear radiation, it is often necessary to provide shielding to reduce exposure to neutron and gamma radiation [60]. Boron carbide possessed strong effects in shielding neutrons, while tungsten was a very effective absorber for gamma-rays. The PEEK/boron carbide composites demonstrated excellent neutron shielding abilities. In addition, the PEEK/boron carbide composites had higher heat deflection temperatures. Under 0.45 MPa load, the heat deflection temperatures of PEEK and PEEK/born carbide (volume ratio of born carbide < 19%) looked almost the same and located near 300 °C [40]. The heat deflection temperatures of PEEK/tungsten composites for shielding gamma-rays was of great significance to the study. All HDT samples (size 80 mm × 10 mm × 4 mm, equivalent to flexural strength sample) were heat-treated. The changes in the heat deflection temperature of PEEK/tungsten composites are shown in Table 4.

**Figure 16:**
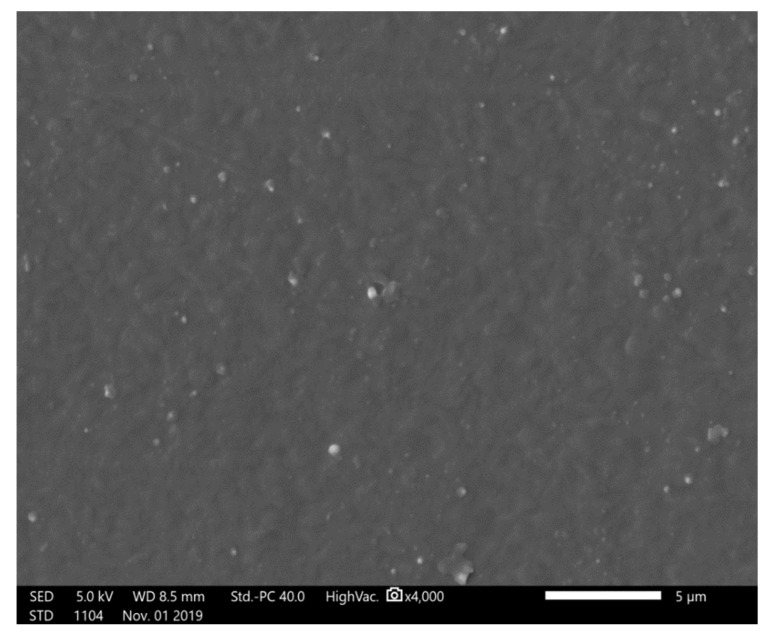
Electron micrograph of PEEK test piece surface (×4000).

The differences in heat deflection temperatures of composite materials with PEEK made of the matrix and tungsten (metal) as a reinforcing agent under the load of 0.45 MPa are presented in Table 4. The heat deflection temperature of 50% PEEK/tungsten composite material was estimated to be 250.0 °C, and that of 70% PEEK/tungsten composite materials was 139.1 °C. The addition of tungsten led to a decrease in the heat deflection temperature of the PEEK composite as a result of the enhanced heat conductivity and specific heat capacity of the material. Under 0.45 MPa load, the increase in temperature led to faster rise in the heat transfer rate of tungsten (metal) than boron carbide (non-metal), and tungsten absorbed more heat than boron carbide. An electron micrograph of the PEEK test piece surface was displayed in Figure 16.

**Figure 17:**
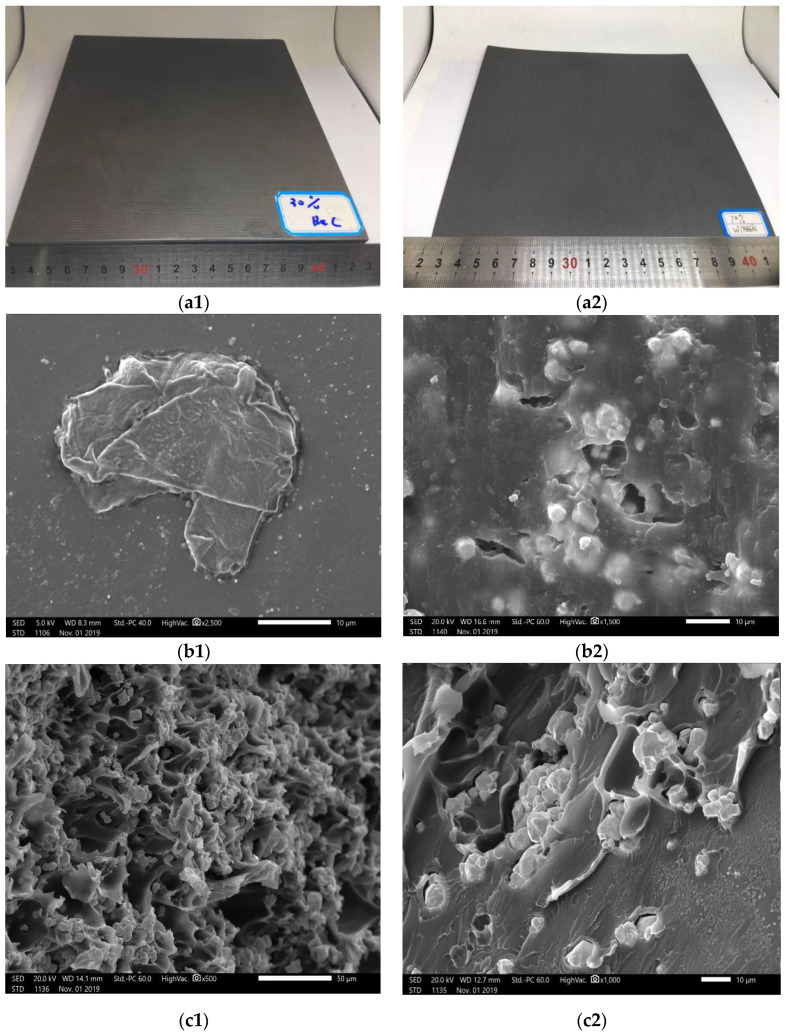
PEEK composite test pieces: (**a1**) 30 wt.% PEEK/boron carbide shielding body, (**a2**) 70 wt.% PEEK/tungsten shielding body, (**b1**) electron micrograph of 30 wt.% PEEK/boron carbide shielding body layer surface (×2500), (**b2**) electron micrograph of 70% PEEK/boron carbide shielding body layer surface (×1500), (**c1**) electron micrograph of broken section of 70 wt.% PEEK/tungsten shielding body (×500), and (**c2**) electron micrograph of broken section of 70 wt.% PEEK/tungsten shielding body (×1000).

The surface of PEEK looked to be flat and free of voids. Pictures of FDM PEEK composite test pieces are presented in Figure 17a1,a2. The magnification of the electron microscope by 2500 times revealed an arrangement of 30% PEEK/boron carbide test piece surface. Agglomerations of boron carbide embedded in PEEK were visible along the free voids (Figure 17b1). At a magnification of 1500 times, obvious voids between the tungsten and PEEK appeared on the layer surface (Figure 17b2). The large heat conductivity of tungsten led to a focused specific heat capacity, fast heat transfer, high heat absorption rate, and large heat transfer energy around tungsten. The shrinkage of PEEK during cooling generated a certain number of gaps. Figure 17c1,c2 display cross-sectional electron micrographs of 70 wt.% PEEK/tungsten test piece. A gap around the aggregated tungsten could clearly be noticed. As tungsten increased, the agglomeration exacerbated the gap, leading to the formation of voids (Figure 17c1,c2). The presence of numerous voids led to a lower heat deflection temperature of PEEK/tungsten composites when compared to PEEK and PEEK/boron carbide composites.

**Figure 18:**
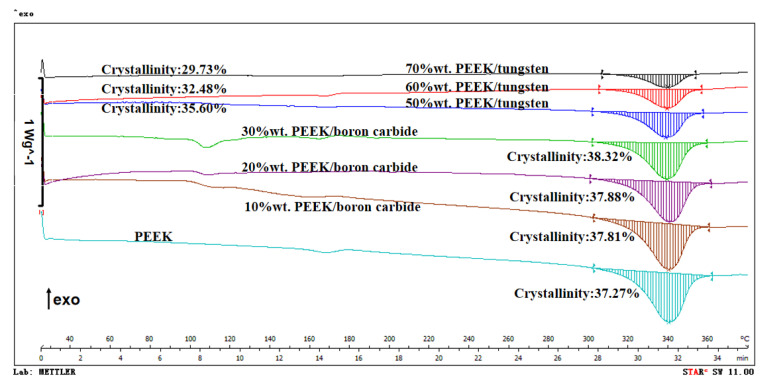
Differential scanning calorimeter (DSC) curves of PEEK, PEEK/boron carbide, and PEEK/tungsten composites (DSC 1 STAR^e^ System, METTLER TOLEDO, Shanghai, China).

Meanwhile, such changes induced differences in crystallinity—a key parameter to characterize the properties of polymers. The respective differential scanning calorimeter (DSC) curves of the PEEK, PEEK/boron carbide, and PEEK/tungsten composites are illustrated in Figure 18. Since the melting of PEEK may induce an endothermic peak, the shaded area in Figure 18 may refer to the crystalline region of PEEK in the composites. The larger crystal area suggested a greater crystallinity and better heat resistance of the materials. The respective crystallinities of PEEK, 10 wt.%, 20 wt.%, and 30 wt.% PEEK/boron carbide composites were recorded as 37.27%, 37.81%, 37.88%, and 38.32%. However, the crystallinity of 50 wt.%, 60 wt.%, and 70 wt.% of the PEEK/tungsten composites appeared significantly lower than those of PEEK and PEEK/boron carbide composites. Compared to PEEK, the crystallinity of 50 wt.%, 60 wt.%, and 70 wt.% of the PEEK/tungsten composites decreased by 4.48%, 12.85%, and 20.23%, respectively. Therefore, the heat deflection temperature of PEEK/tungsten composites diminished as tungsten content increased.

### 3.2. Gamma-Ray Shielding Performance

In the experiment, test pieces were heat treated, and attenuation characteristics of the new PEEK/tungsten shielding materials are listed in Table 5. The values may further explain the linear attenuation coefficient and half-absorption thickness. Note that sample #0 represented pure PEEK material, and #1, #2 and #3 refer to, respectively, 50 wt.%, 60 wt.% and 70 wt.% PEEK/tungsten composite materials. Note that two sources of energy (^137^Cs and ^60^Co) were used in the experiments.

**Figure 19:**
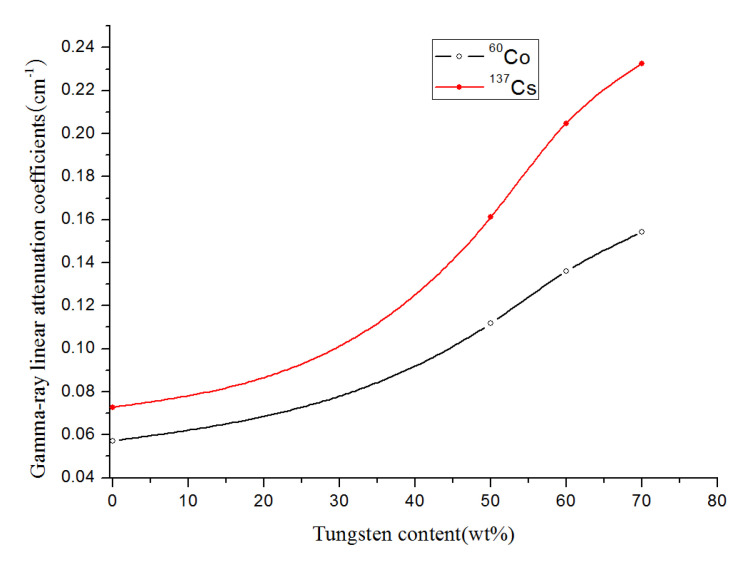
Comparison of attenuation characteristics of ^137^C_S_ and ^60^Co sources.

In Table 5, the linear absorption coefficient of sample #0 at low-energy ^137^Cs source was determined as 0.0728 cm^−1^. Compared to #0 test piece, the addition of tungsten relatively increased the linear absorption coefficients of samples #1, #2 and #3 by 54.81%, 64.45% and 68.70%, respectively. As the tungsten content rose, the shielding effect of the composites also enhanced. At a high-energy ^60^Co source, the linear absorption coefficients of samples #1, #2 and #3 incremented by 48.84%, 57.91% and 62.91%, respectively, when compared to the #0 material test piece. The relationships between the change in tungsten content of PEEK/tungsten composite materials and linear absorption coefficient under the two energy radiations are provided in Figure 19.

**Figure 20:**
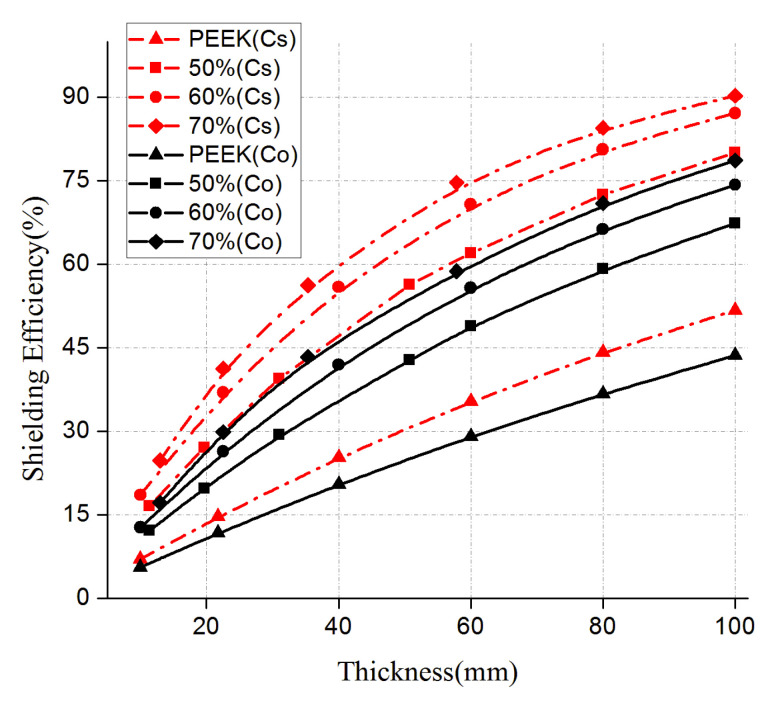
Gamma-ray shielding properties of PEEK composites with different thicknesses and different tungsten contents.

The FDM 3D printed shielding test pieces displayed good shielding effects at ^137^C_S_ and ^60^Co sources (Figure 19). Higher tungsten contents led to a better shielding effect. As for test pieces containing the same tungsten content, the linear attenuation absorption coefficient obtained at ^137^Cs was superior to that at ^60^Co, indicating a better shielding effect of the new materials against low-energy gamma-rays (^137^Cs) when compared to high-energy gamma-rays (^60^Co). The shielding properties of PEEK composites with different thicknesses and tungsten contents are compiled in Figure 20. The black and red curves represent the gamma-ray shielding effects under ^60^Co and ^137^Cs sources, respectively.

The ^60^Co and ^137^Cs sources gamma-ray shielding rate of the #0 material with a thickness of 10mm was estimated to be 5.56% for both types of radiation. At a thickness of 100 mm, the ^60^Co source gamma-ray shielding rate increased to 43.60%, and that of ^137^Cs was 51.71%. Thus, PEEK showed a certain shielding ability of gamma-rays, and increasing the thickness effectively improved the shielding efficiency. For the #1 composite material with a thickness of 100mm, the ^60^Co source gamma-ray shielding rate was calculated as 67.30%, and that of ^137^Cs source was 80.03%. For the #2 composite material with a thickness of 100 mm, the ^60^Co and ^137^Cs source gamma-ray shielding rates were estimated to 74.25% and 87.10%, respectively. The increase in the thickness of the #3 sample to 100 mm led to improved ^60^Co and ^137^Cs source gamma-ray shielding rates (78.60% and 90.23%, respectively). Therefore, the gamma-ray shielding effect of test pieces with the same thickness was proportional to the tungsten content present in the composite materials. The addition of more tungsten led to better shielding effects. Using the same material, the shielding effect varied proportional with the thickness. In other words, greater thicknesses led to better shielding effects.

Since the inelastic scattering and radiation capture of neutrons may generate secondary gamma-rays, a multi-layered hetero-structure should be designed for shielding neutron radiation. The PEEK/tungsten was placed closer to the neutron source and might reduce neutrons energy. The PEEK/boron carbide composite material was designed at a remote location to absorb neutrons. Such a structure may effectively shield neutrons and secondary gamma-ray radiations.

## 4. Conclusions

A novel type of composite material for shielding of gamma-rays was successfully prepared. The shielding test pieces were processed by FDM 3D printing and tested against gamma-rays. The following conclusions could be drawn:

(1) Gamma-ray shielding was properly achieved by PEEK/tungsten composite materials prepared by FDM 3D printing. The new shielding materials showed low porosity, dense structure, uniform tungsten dispersion, simple flow processing, and a short cycle. These features were appropriate for solving problems encountered by traditional PEEK and PEEK composite materials, such as difficult processing, high cost, and the presence of many defects.
(2) The mechanical properties of the new shielding materials were tested and the data revealed excellent tensile and flexural strengths of PEEK/tungsten composite materials. After heat-treatment, the tensile strengths of 50 wt.%, 60 wt.% and 70 wt.% PEEK composites increased by 33.51%, 27.52% and 30.60%, respectively. The flexural strengths also improved by 33.28%, 34.81%, and 41.29%, respectively. Hence, heat-treatment was effective for improving the mechanical properties.
(3) The PEEK/tungsten composite materials displayed good heat deflection temperatures. The values of the heat deflection temperature of 50 wt.%, 60 wt.% and 70 wt.% PEEK/tungsten composite materials under 0.45MPa were calculated as 250 °C, 211.4 °C and 139.1 °C, respectively. The addition of tungsten led to a significant decline in the heat deflection temperature, which reduced by 14.99%, 28.12%, and 52.70% for the above-mentioned materials when compared to the heat deflection temperature of PEEK. Metal and non-metal PEEK composite materials showed very different heat deflection temperatures. Therefore, multi-layered heterostructures should be designed for shielding nuclear radiation. The high temperature resistant of PEEK/boron carbide was close to the radiation source and absorbed heat neutrons. The PEEK/tungsten composite material was designed far away from the source to shield gamma-rays produced by neutron radiation.
(4) The irradiation by two different energy rays (^137^Cs and ^60^Co) revealed PEEK/tungsten composites to possess more effective shielding abilities toward low than high energy gamma-rays. For shielding test bodies with the same thickness, the shielding rates enhanced exponentially with tungsten content. More tungsten led to greater linear attenuation coefficients of test pieces and improved shielding effects. The shielding efficiencies of 100 mm PEEK, 50 wt.%, 60 wt.% and 70 wt.% PEEK/tungsten composites irradiated by ^137^Cs sources were estimated to be 43.60%, 67.30%, 74.25% and 78.60%, respectively. The shielding efficiencies of 100mm PEEK, 50 wt.%, 60 wt.% and 70 wt.% PEEK/tungsten composite materials irradiated by ^60^Co source were recorded as 51.71%, 80.03%, 87.10% and 90.23%, respectively. At the same tungsten content, the shielding efficiency increased with the thickness of the shielding test body.
(5) In sum, further studies of the novel PEEK/tungsten shielding materials and FDM processing technology are required to gain more insights. Future work will focus on the integration of simulations, structural design, 3D printing, and property testing. Additionally, multi-layered hetero-structures with optimal mechanical properties and shielding performances for multiple rays should be designed for complex environments.

## Figures and Tables

**Table 1:** Material ratios of PEEK/tungsten composite materials.

Material Number	Name of Shielding Material	Mass Ratio of Tungsten (wt.%)	Volume Ratio of Tungsten (vol%)
#0	Pure PEEK	0	0
#1	50 wt.% PEEK/tungsten composite material	50	6.30
#2	60 wt.% PEEK/tungsten composite material	60	9.15
#3	70 wt.% PEEK/tungsten composite material	70	13.55

**Table 2:** The compactness values of PEEK and PEEK composites.

Material Number	Name of Shielding Material	Actual Density of Wire (g/cm^3^)	Theoretical Density of Wire (g/cm^3^)	Compactness of Wire (%)
#0	Pure PEEK	1.300	1.300	100
#1	50 wt.% PEEK/tungsten composite material	2.422	2.520	96.11
#2	60 wt.% PEEK/tungsten composite material	2.903	3.050	95.18
#3	70 wt.% PEEK/tungsten composite material	3.674	3.870	94.94

**Table 3:** FDM processing parameters of PEEK/tungsten composite materials.

Material Number	Nozzle Diameter (mm)	Printing Speed (mm/s)	Nozzle Temperature (°C)	Filling Line Width (mm)	Filling Line Overlapping Ratio (%)	Lapping Width (mm)	Height (mm)
#0	0.4	20	410 °C	0.4	10	0.04	0.2
#1	0.4	20	420 °C	0.4	15	0.06	0.2
#2	0.4	20	425 °C	0.4	15	0.06	0.2
#3	0.4	20	430 °C	0.4	15	0.06	0.2

**Table 4:** Heat deflection temperatures of new shielding materials.

Name	Volume Ratio of Tungsten (vol%)	Heat Deflection Temperature (°C)
50 wt.% PEEK/tungsten composite	6.52 (Tungsten)	250.0
60 wt.% PEEK/tungsten composite	9.47 (Tungsten)	211.4
70 wt.% PEEK/tungsten composite	14.00 (Tungsten)	139.1

**Table 5:** Attenuation characteristics of the new gamma-ray shielding PEEK/tungsten composites.

Materials Number	Mass Ratio of Tungsten (wt.%)	Volume Ratio of Tungsten (vol%)	^137^Cs (0.662 MeV)		^60^Co (1.25 MeV)	
			μ (cm^−1^)	d_½_ (cm)	μ (cm^−1^)	d_½_ (cm)
#0	0	0	0.0728	9.5192	0.0572	12.1154
#1	50	6.52	0.1611	4.3017	0.1118	6.1986
#2	60	9.47	0.2048	3.3838	0.1359	5.0993
#3	70	14.00	0.2326	2.9794	0.1542	4.4942
